# Incidental gastrointestinal ^18^F-Fluorodeoxyglucose uptake associated with lung cancer

**DOI:** 10.1186/s12890-015-0152-6

**Published:** 2015-12-02

**Authors:** Juliette Vella-Boucaud, Dimitri Papathanassiou, Olivier Bouche, Alain Prevost, Thibault Lestra, Sandra Dury, Hervé Vallerand, Jeanne-Marie Perotin, Claire Launois, Louis Boissiere, Mathilde Brasseur, François Lebargy, Gaëtan Deslee

**Affiliations:** Service des Maladies Respiratoires, Hôpital Maison Blanche, CHU de Reims, 45 rue Cognacq Jay, 51092, Reims, Cedex France; Service de Médecine Nucléaire, Institut Jean Godinot, Centre de Lutte Contre le Cancer à Reims, Reims, France; Service d’Hépato-Gastro-Entérologie et Cancérologie Digestive, Hôpital Robert Debré, CHU de Reims, Reims, France; Unité de Médecine Ambulatoire Cancérologie Hématologie, Hôpital Robert Debré, CHU Reims, Reims, France; Service d’Oncologie Médicale, Institut Jean Godinot, Centre de Lutte Contre le Cancer à Reims, Reims, France

**Keywords:** Gastrointestinal Tract, Incidental Findings, Lung Neoplasms, Fluorodeoxyglucose F18, Positron Emission Tomography

## Abstract

**Background:**

F-Fluorodeoxyglucose positron emission tomography/computed tomography (^18^F-FDG PET/CT) is increasingly used for the initial staging and restaging of lung cancer. Incidental gastrointestinal findings are often observed on ^18^F-FDG PET/CT. The objective of this study was to assess incidental ^18^F-FDG uptake by the gastrointestinal tract (GIT) in patients with lung cancer.

**Methods:**

Two hundred thirty consecutive ^18^F-FDG PET/CT examinations performed for lung cancer over a 3-year period were retrospectively reviewed for the presence of incidental FDG uptake in the GIT. The charts of patients with positive FDG uptake were then reviewed and analysed to determine the GIT uptake sites, the standardized uptake value (SUV) max and the final clinical diagnosis.

**Results:**

Fifty-two patients (52/230, 23 %) demonstrated incidental FDG uptake in the GIT. Thirty-three patients (63.5 %) had diffuse uptake (oesophagus, *n* = 2, colon, *n* = 31) and 19 patients (36.5 %) had focal uptake (oesophagus, *n* = 1, small bowel, *n* = 1, ascending colon, *n* = 5, descending colon, *n* = 4, sigmoid, *n* = 4, rectum, *n* = 3, and anal margin, *n* = 1). Twelve of the 52 patients with GIT uptake were further investigated, revealing, a diagnosis of malignancy in 4 patients with focal FDG uptake. No significant differences in mean SUVmax were observed between patients with malignant and benign GIT diseases.

**Conclusion:**

This study demonstrates a high incidence of FDG uptake in the GIT associated with lung cancer. Focal GIT uptake was frequently associated with malignant disease. These results suggest that further GIT investigations should be performed in patients with focal GIT uptake.

## Background

Lung cancer is the most common cause of cancer-related deaths worldwide. The best chance for cure in these patients is complete surgical resection. However, the overall 5-year survival rate does not exceed 20 % [1]. In view of the high incidence of metastatic disease in lung cancer, accurate tumour staging is an essential and critical step for the choice of optimal treatment strategies. TNM stage is the most important prognostic factor to guide treatment decisions [2, 3]. Staging typically includes imaging techniques such as computed tomography (CT), magnetic resonance imaging (MRI), and bone scintigraphy. Positron emission tomography, using the glucose analogue ^18^F-fluorodeoxyglucose (^18^F-FDG-PET/CT), is increasingly used for initial staging, restaging and monitoring of response to treatment of lung cancer [4–10]. It has been demonstrated to be an effective tool, providing more precise and reliable assessment than conventional methods and it has become the standard of care for these purposes [11].

As a result of the increased availability of ^18^F-FDG PET/CT for patients with malignancy, unexpected ^18^F-FDG uptake has been identified in a variety of sites, including the gastrointestinal tract (GIT) [12, 14]. Incidental gastrointestinal uptake is found in about 3 % of patients undergoing evaluation for non-gastrointestinal diseases [15]. The mechanism of increased ^18^F-FDG uptake in the GIT is unclear, but may be related to physiological, inflammatory, benign or malignant processes*.* Two different patterns of GIT ^18^F-FDG uptake have been defined on PET/CT examination: focal and diffuse [16]. While diffuse ^18^F-FDG uptake is generally correlated with a physiological uptake, focal ^18^F-FDG uptake can be associated with a detectable disease such as primary tumour, sites of unusual metastatic spread or premalignant lesions. Previous reports have evaluated the incidence and clinical significance of incidental gastrointestinal ^18^F-FDG uptake and concluded that this finding deserves further appropriate investigation [12, 23].

The objectives of this retrospective study were to evaluate incidental ^18^F-FDG uptake in the GIT observed in a patient population assessed by PET/CT for staging or restaging of lung cancer and to assess the clinical significance of these unexpected findings.

## Methods

### Patients

We performed a retrospective review of 230 consecutive patients who underwent ^18^F-FDG PET/CT for lung cancer staging or restaging between January 2011 and January 2014 in the Reims Regional Cancer Centre (Jean Godinot Institute, IJG). When several ^18^F-FDG PET/CT examinations were performed in the same patient, only the first ^18^F-FDG PET/CT was analysed. This study was approved by the Reims University Hospital Institutional Review Board (IRB).

Patients with incidental FDG uptake in the gastrointestinal tract (GIT) were identified by reviewing PET/CT reports. One person (JVB) assessed all PET/CT reports. Incidental GIT FDG uptake was described according to two patterns, focal and diffuse uptake compared to background activity assessed by the nuclear medicine physicians. All cases of incidental FDG uptake in the GIT were analysed. The following data were extracted for all patients (*n* = 230): demographic data, indications for ^18^F-FDG PET/CT, histology and clinical stage of lung cancer, and extrathoracic FDG-uptake with organ localization. In patients with GIT FDG uptake, data concerning ^18^F-FDG PET/CT, GIT investigations and final diagnosis of incidental GIT findings were also extracted.

### ^18^F-FDG PET/CT protocol

PET examinations were performed with a Gemini-Dual (Philips®) PET/CT camera, according to a standard protocol for cancer imaging. Briefly, PET/CT acquisition was performed as follows: 3 min in each bed position covering the body between the pelvis and the base of the skull one hour after peripheral intravenous injection of 5 MBq/kg of ^18^F-FDG in patients fasted for at least 6 h before the examination, and low-dose unenhanced CT (standard acquisition parameters: 100 mAs, 120 kV tube voltage, 1.5 pitch, 6.5 mm slice thickness). A nuclear medicine physician reviewed all ^18^F-FDG PET/CT examinations demonstrating GIT uptake (52/230 PET/CT were assessed by one nuclear medicine physician (DP)) to determine the maximum standardized uptake value (SUVmax) of all patients. SUVpeak (mean of SUV in a spherical volume of interest of approximately 1 mL in a position that provided the maximal average in the area of uptake) was also obtained for patients with focal FDG uptake. Two additional measurements of the metabolic volume using different definitions of volumes of interest with varying isocontours (3D isocontour at 50 % and 41 % of the maximum pixel value) were also calculated.

### GIT

The GIT was divided into the oesophagus, stomach, small bowel, colon, rectum and anal margin. In our study, the final diagnoses were classified as *i) “*malignant disease confirmed” when the malignant disease was confirmed by histopathology, *ii)* “high probability of malignant disease” when the malignant disease was not confirmed by endoscopy and histopathology but was highly probable based on follow-up data including clinical findings, CT, new FDG-PET/CT and/or endoscopy, *iii) “*benign disease confirmed” when endoscopy was normal, and *iv) “*high probability of benign disease” when endoscopy was not performed but no sign of malignant GIT disease was detected on follow-up data including clinical findings, CT, new FDG-PET/CT and/or endoscopy.

### Statistical analysis

Quantitative variables are expressed as mean ± standard deviation (SD) and qualitative variables are expressed as number and percentage. Student test and Chi-square test were performed to compare patient characteristics between positive or negative GIT FDG uptake. Mann–Whitney test was performed to compare SUV between the 4 groups of diagnosis. A p value < 0.05 was considered statistically significant. All analyses were performed using Epi Info version 7.0.

## Results

### Patient characteristics

**Fig. 1:**
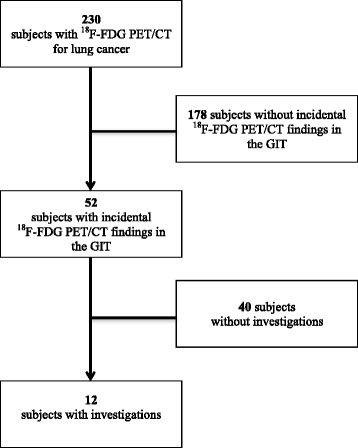
Flowchart of the retrospective study

^18^F-FDG PET/CT was performed in 230 patients with lung cancer between January 2011 and January 2014. Incidental FDG uptake in the GIT was reported in 52 of these patients, representing a prevalence of 22.6 % (Fig. 1). Patient characteristics are described in Table 1. The mean age was 66 ± 9 years in the PET/CT GIT positive group and 62 ± 10 years in the PET/CT GIT negative group (*p* < 0.05). Both groups comprised a majority of men, and the primary lung cancer histology was adenocarcinoma. The most common indication for PET/CT was staging and the leading extrathoracic site of uptake on PET-CT was bone. Seventeen of the 52 patients (32.7 %) had diabetes and 13/17 (76.5 %) patients were treated with oral antidiabetic drugs. All patients treated with oral antidiabetic drugs presented diffuse GIT uptake.

**Table 1:** Patient characteristics

	Subjects with PET/CT for lung cancer	PET/CT GIT	PET/CT GIT
		Positive group	Negative group
	*n* = 230	*n* = 52	*n* = 178
Age	63 ± 10	66 ± 9	62 ± 10*
Gender			
Male	170 (74 %)	39 (75 %)	131 (74 %)
Female	60 (26 %)	13 (25 %)	47 (26 %)
TNM stage			
I	29 (12.6 %)	7 (13.5 %)	22 (12 %)
II	29 (12.6 %)	11 (21 %)	18 (10 %)*
III	81 (35.2 %)	12 (23 %)	69 (39 %)*
IV	91 (39.6 %)	22 (42.5 %)	69 (39 %)
Histology			
Adenocarcinoma	110 (48 %)	22 (42 %)	88 (49 %)
Squamous cell carcinoma	73 (31.5 %)	17 (33 %)	56 (31 %)
Small cell lung carcinoma	24 (10.5 %)	7 (13.5 %)	17 (10 %)
Others	23 (10 %)	6 (11.5 %)	17 (10 %)
Indications			
Staging	196 (85 %)	46 (88.5 %)	150 (84 %)
Restaging	34 (15 %)	6 (11.5 %)	28 (16 %)
Extrathoracic Hypermetabolic state	125 (54 %)	52 (100 %)	73 (41 %)
Adrenal glands	18 (7.8 %)	8 (15 %)	10 (6 %)*
Bone	45 (20 %)	11 (21 %)	34 (19 %)
Brain	5 (2 %)	2 (4 %)	3 (2 %)
Head and neck	24 (10.5 %)	4 (8 %)	22 (11 %)
Muscle	10 (4.3 %)	6 (11.5 %)	4 (2 %)
Thyroid	3 (1.3 %)	0	3 (2 %)
Others	39 (17 %)	5 (10 %)	34 (19 %)

Data are expressed as number with percentages in parentheses (%) or mean ± SD

* *p* value < 0.05

### GIT findings

The characteristics and sites of incidental FDG uptake in the PET/CT GIT positive group were described according to two patterns, focal and diffuse uptake (Table 2). Thirty-three (63.5 %) of these patients had diffuse uptake and 19 (36.5 %) had focal uptake. Among the 33 patients with diffuse ^18^F-FDG uptake, uptake was observed in the oesophagus in 2 cases and in the colon in 31 cases. Among the 19 patients with focal FDG-uptake, uptake was observed in the oesophagus in 1 case, in the small bowel in 1 case, in the colon in 13 cases (5 in the ascending colon, 4 in the descending colon, 4 in the sigmoid), in the rectum in 3 cases and in the anal margin in 1 case. The colon was the most common site of FDG uptake in both groups.

**Table 2:** Characteristics and sites of incidental GIT ^18^fluorodeoxyglucose uptake on PET/CT images

	Diffuse uptake *n* = 33	Focal uptake *n* = 19
Location		
Oesophagus	2 (6 %)	1 (5.3 %)
Stomach	0	0
Small bowel	0	1 (5.3 %)
Colon	31 (94 %)	13 (68.5 %)
Rectum	0	3 (16 %)
Anal margin	0	1 (5.3 %)
SUVmax	8.7 ± 5.1	6.4 ± 3.8

Data are expressed as mean ± SD or number (%)

SUVmax = maximum standardized uptake value

### Investigations and diagnosis

Twelve of the 52 patients with incidental GIT findings (diffuse uptake, *n* = 33; focal uptake, *n* = 19) were investigated by colonoscopy (*n* = 8), gastroscopy (*n* = 3), and abdomen CT (*n* = 1) (Table 3). Investigations were performed in 9 patients with focal GIT uptake (47 %), and 3 patients with diffuse GIT uptake (9 %).

**Table 3:** Investigations and diagnosis of incidental gastrointestinal tract findings

	Focal uptake (*n* = 19)	Diffuse uptake (*n* = 33)	Total (*n* = 52)
Investigations	9	3	12 (23 %)
Colonoscopy	7	1	8 (67 %)
Gastroscopy	1	2	3 (25 %)
CT abdomen	1	0	1 (8 %)
Diagnosis			
Malignant disease confirmed	4	0	4 (8 %)
High probability of malignant disease	5	0	5 (9.5 %)
Benign disease confirmed	4	3	7 (13.5 %)
High probability of benign disease	6	30	36 (69 %)

Data are expressed as number (%)

**Fig. 2:**
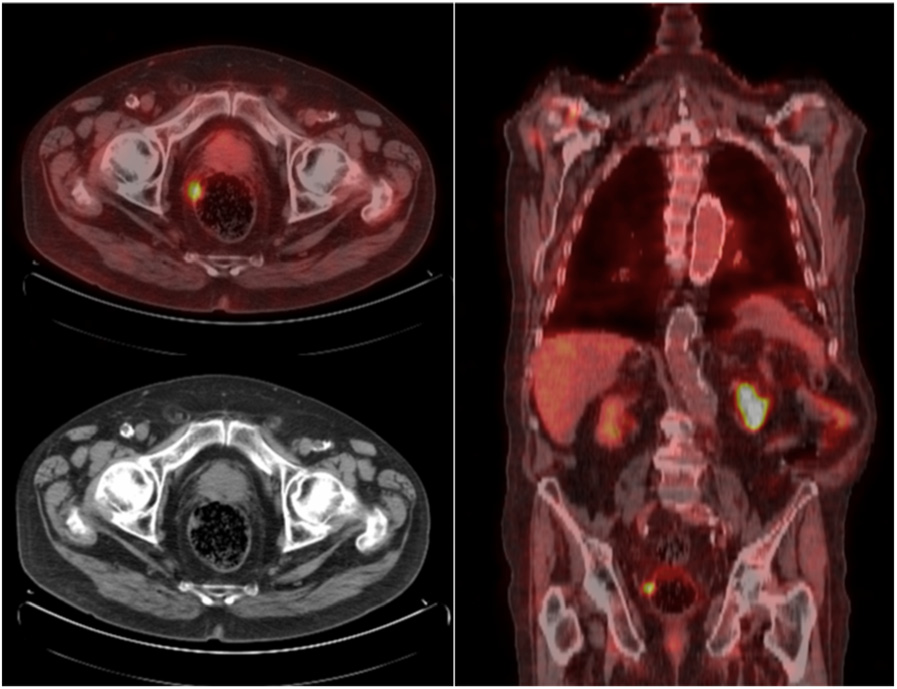
Coronal and axial positron emission tomography scan showing unexpected focal rectal uptake. The final diagnosis was rectal adenocarcinoma

Of the 9 patients with focal uptake who were investigated, 4 patients were diagnosed with confirmed malignant GIT lesions (colon adenocarcinoma, *n* = 2; rectal adenocarcinoma, *n* = 1; oesophageal metastasis of lung cancer, *n* = 1) (Table 3, Fig. 2). One patient had a high probability of malignant disease (colon carcinoma) based on a suspicious lesion on PET-CT confirmed by further abdominal CT scan, but no further investigations were performed because of the clinical status of this patient with metastatic lung cancer. Two patients had benign diseases, including one case of diverticulitis, and one case of faecal impaction. Colonoscopy was strictly normal in two patients, with no signs of GIT disease on follow-up. No additional diagnostic procedure was performed in 10 patients with focal FDG uptake. The reason for not performing additional GIT assessments in patients with focal FDG uptake was metastatic lung cancer with poor functional status despite the high probability of malignant GIT disease based on PET-CT findings (*n* = 4), or a high probability of benign GIT disease based on benign PET scan findings with no abnormality on abdominal CT scan and subsequent clinical follow-up (*n* = 6).

Two of the 3 patients investigated for diffuse FDG uptake presented signs of erosive oesophagitis and one patient had no abnormality on colonoscopy, performed in view of a family history of colon cancer. Of note, none of the 33 patients with diffuse uptake developed malignant gastrointestinal disease on the basis of subsequent clinical follow-up.

Overall, the final GIT diagnoses associated with GIT uptake was malignant disease confirmed (*n* = 4, 8 %), high probability of malignant disease (*n* = 5, 9.5 %), benign disease confirmed (*n* = 7, 13.5 %) and high probability of benign disease (*n* = 36, 69 %). The GIT pathological findings in patients with focal uptake modified the treatment strategy for only one patient, who was treated by surgery for two independent malignant lesions in the lung and colon, with no evidence of metastasis.

### SUV

Mean SUVmax values were 8.7 ± 5.1 for diffuse FDG uptake, 6.4 ± 3.8 for focal FDG uptake, 7.05 ± 3 for the malignant disease confirmed group, 7 ± 3 for the high probability of malignant disease group, 6.5 ± 6.1 for the benign disease confirmed group and 8.3 ± 5 for the high probability of benign disease group. No statistically significant differences in terms of mean SUVmax values were observed between focal and diffuse FDG uptake groups (*p* = 0.19) or between the 4 diagnosis groups (*p* = 0.69). No statistically significant difference in terms of mean SUVpeak, SUV50 and SUV41 was observed between the 4 diagnosis groups among patients with focal FDG uptake (Table 4). GIT abnormalities were identified on CT scan in 7 (37 %) of the 19 patients with focal FDG-uptake (Table 4).

**Table 4:** Standardized uptake values of focal FDG uptake in the gastrointestinal tract and lesions detected by CT scan

	Malignant disease confirmed	High probability of malignant disease	Benign disease confirmed	High probability of benign disease
	*n* = 4	*n* = 5	*n* = 4	*n* = 6
SUVmax	7.05 ± 3	7 ± 3	7.7 ± 7.3	4.7 ± 0.7
SUVpeak	5.8 ± 3	5.8 ± 2.5	6.5 ± 5.3	3.5 ± 0.7
Volume 50 %	5.8 ± 4.6	6.2 ± 2.9	6.5 ± 3.3	4.3 ± 4.8
Volume 41 %	7.5 ± 5.5	8.7 ± 3.9	8.6 ± 7.8	6 ± 5.3
Lesion on CT scan	2 (50 %)	2 (40 %)	1 (25 %)	2 (33 %)

Data are expressed as number with percentages in parentheses (%) or mean ± SD

Volume 50 % et 41 %: 3D isocontour at 50 % and 41 % of the maximum pixel value

## Discussion

In this series, 23 % of patients (52/230) assessed by PET/CT imaging for lung cancer exhibited incidental GIT FDG-uptake. Thirty-three (63.5 %) of these patients presented diffuse uptake and 19 (36.5 %) presented focal uptake. Further investigations were performed in 12 of the 52 patients with incidental GIT findings. A diagnosis of malignancy was established in 4 of the 12 patients with focal uptake. No significant differences in mean SUVmax were observed between the malignant disease confirmed, high probability of malignant disease, benign disease confirmed and high probability of benign disease groups.

With the increasing use of PET/CT imaging, the management dilemma posed by patients with incidental PET/CT findings unrelated to the primary malignancy is likely to become an increasingly common situation. In our study, 23 % of cases presented incidental GIT FDG uptake. This prevalence is higher than that reported in other studies performed in all types of cancer with GIT FDG uptake rates ranging from 1.3 to 3 % [15, 20].

The gastrointestinal tract is a common site of physiological and incidental FDG uptake [12-23]. Focal tracer uptake is commonly seen at the gastro-oesophageal junction with moderate uptake in the stomach, low-intensity uptake in the small bowel, and diffuse or focal uptake in the colon. Higher uptake has been described in the caecum and ascending colon, presumably due to the higher concentration of lymphocytes [16, 26]. Other mechanisms for this physiological activity have been attributed to uptake by muscular peristaltic activity (mainly in the bowel), swallowed secretions or excretion and intraluminal concentration of FDG [27, 28]. Increased FDG uptake can also be related to inflammation such as enterocolitis, pseudomembranous colitis, inflammatory bowel disease, and ulcerative colitis [20, 29–31], or iatrogenic causes such as oral antidiabetic drugs. In this study, all patients taking oral antidiabetic drugs presented diffuse GIT uptake and no additional investigation was performed. It should be noted that all cases of positive GIT uptake were reviewed by a nuclear medicine physician, allowing the GIT FDG uptake results to be considered as robust. In previous studies, two patterns of GIT FDG uptake have been described, diffuse and focal uptake. In our study, 33/52 patients presented diffuse uptake. Three patients with diffuse uptake, including 2 patients with oesophageal FDG uptake, were further investigated. As described in the literature, no case of malignancy was found in patients with diffuse uptake in our study [17]. Focal GIT uptake must be interpreted cautiously and further investigations are highly recommended in these patients. In our study, 9/19 patients were investigated following the detection of focal FDG uptake. Note that the prevalence of focal FDG uptake in our study was higher than that reported in other studies including all types of cancer [24]. Previous studies have shown that the majority of foci of incidental FDG uptake in the GIT, especially in the colon and rectum, were confirmed to be due to significant pathology [13, 15, 18, 20], while only a small proportion of sites in the rest of the GIT was attributed to malignancy. A significant GIT disease (cancer, diverticulitis, erosive oesophagitis, ulcerative colitis) was diagnosed in 58 % of patients undergoing further GIT investigations in our study. Fifty-five percent of patients with focal FDG uptake investigated in our study presented malignant disease or a high probability of malignant disease. Similar results have been reported in two recent studies with colon cancer diagnosed in 33 % and 35 % of patients with incidental GIT FDG PET/CT uptake associated with pulmonary nodules [24, 25]. These findings are consistent with most published studies, thereby emphasizing the need for further GIT investigations to confirm focal FDG uptake, especially in the colon. Chopra et al. [38], in a retrospective analysis of PET/CT scan reports of patients with lung cancer, showed that the large bowel was the commonest site for incidental findings and detected malignant or premalignant abnormalities in 76 % of patients undergoing colonoscopy.

Gastrointestinal cancer and lung cancer present a number of risk factors in common, such as age and smoking. Age remains the most significant risk factor for colorectal cancer and most patients with lung cancer are also elderly. Smoking is a known risk factor for stomach and oesophagus cancer and Giovannucci et al. recently speculated that smoking may be the potential cause of up to one in five colorectal cancers [32].

Colorectal cancer was detected incidentally in 3 of a series of 230 (1.3 %) patients, which is higher than the rate observed in various FDG PET/CT cancer screening studies, such as that reported by Chen et al. with a colorectal cancer incidence of 0.19 % (6/3210), Terauchi et al. with an incidence of 0.96 % (28/2911), and Purandare et al. with an incidence of 0.055 % (5/9000) [33–35] probably related to potential common risk factors between colon cancer and lung cancer in our series.

No significant difference in SUVmax was observed the two groups with focal or diffuse incidental GIT uptake, and no significant difference in SUVmax, SUVpeak, SUV50 and SUV41 was observed between the four diagnosis groups, confirming previously published data [13, 14, 18–20, 23, 35, 36]. In our study, the mean SUVmax of diffuse FDG uptake was slightly higher than the mean SUVmax of focal FDG uptake, although diffuse FDG uptake was generally associated with a benign diagnosis. The SUVmax value was not significantly different between malignant and benign lesions and no cut-off value was identified for SUVmax in our study [23–37].

Our study presents a number of limitations. First, the study is limited by its retrospective design. Second, a relatively small number of patients was included in our study, and a small number of patients were considered for further GIT investigations, mainly due to the presence of metastatic disease. Of note, our population was selected on the basis of a final diagnosis of lung cancer, excluding patients with benign or secondary pulmonary nodules [24]. Despite these limitations, this study demonstrates a high incidence of GIT PET/CT uptake associated with lung cancer, and found that a focal pattern of FDG uptake was frequently associated with malignant disease or a high probability of malignant disease in the GIT.

## Conclusion

In conclusion, positive GIT findings on FDG-PET/CT imaging during staging or restaging of lung cancer may constitute a clinically significant finding, especially when uptake is focal and localized in the colon or rectum. The high incidence of GIT cancer associated with lung cancer in our study indicates the need for careful analysis of PET/CT imaging and further GIT investigations in patients with focal GIT FDG uptake.

## Abbreviations

^18^F-FDG: 18-F-Fluorodeoxyglucose
^18^F-FDG PET/CT: 18-F-Fluorodeoxyglucose positron emission tomography/computed tomography
CT: Computed tomography
GIT: Gastrointestinal tract
MRI: Magnetic resonance imaging
SUV: Standardized uptake value
SUV max: Maximum standardized uptake value.
